# Supplementary material for: The epidemiology of atopic dermatitis in older adults: A population-based study in the United Kingdom
Source: PLoS One. 2021 Oct 6;16(10):e0258219. doi: 10.1371/journal.pone.0258219 (PMC8494374; doi:10.1371/journal.pone.0258219)

S1 Fig. Correcting for ascertainment bias. Local polynomial smoothed plots with shading indicating the 95% CIs generated from yearly cross-sectional calculations of the prevalence of atopic dermatitis from ages 0 to 99 within a 1% random sample of the population. In a correction for ascertainment bias, the prevalence of atopic dermatitis by year of age was restricted to those who had at least one medical code corresponding to a physician's visit during that year.

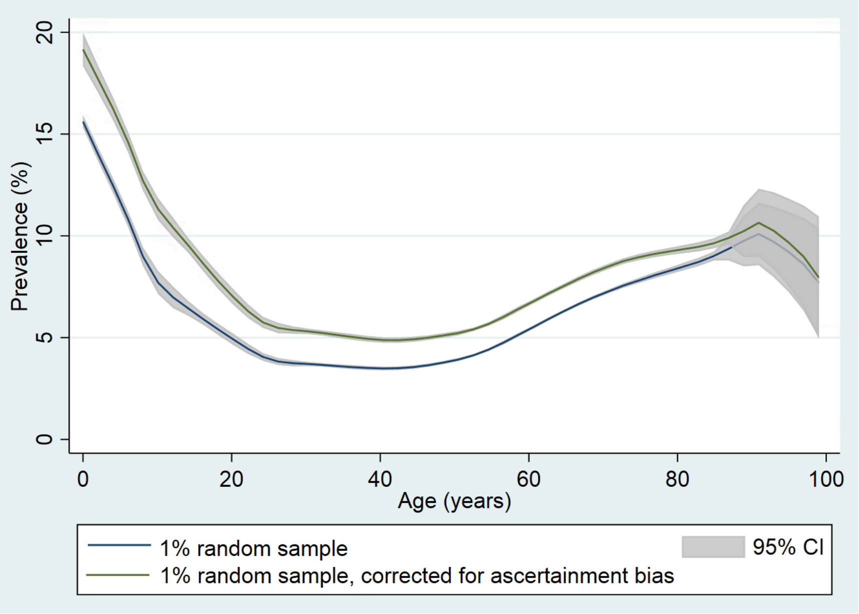

Supplement: S1 Fig — Local polynomial smoothed plots with shading indicating the 95% CIs generated from yearly cross-sectional calculations of the prevalence of atopic dermatitis from ages 0 to 99 within a 1% random sample of the population. In a correction for ascertainment bias, the prevalence of atopic dermatitis by year of age was restricted to those who had at least one medical code corresponding to a physician’s visit during that year. (PDF) [file pone.0258219.s001.pdf]
