# Supplementary material for: The epidemiology of atopic dermatitis in older adults: A population-based study in the United Kingdom
Source: PLoS One. 2021 Oct 6;16(10):e0258219. doi: 10.1371/journal.pone.0258219 (PMC8494374; doi:10.1371/journal.pone.0258219)

S2 Fig. Prevalence of atopic dermatitis across age in the 2005-2006 NHANES. Local polynomial smoothed plots with shading indicating the 95% CIs generated from yearly cross-sectional calculations of the percent with prevalent atopic dermatitis during the past year from ages 0 to 85+. A) Three definitions representing prevalence of atopic dermatitis were formed by separate combinations of survey questions relevant to atopic dermatitis B) Prevalence of atopic dermatitis by sex using Definition 1 and C) Prevalence of atopic dermatitis by income using Poverty Income Ratio (PIR) using Definition 1.

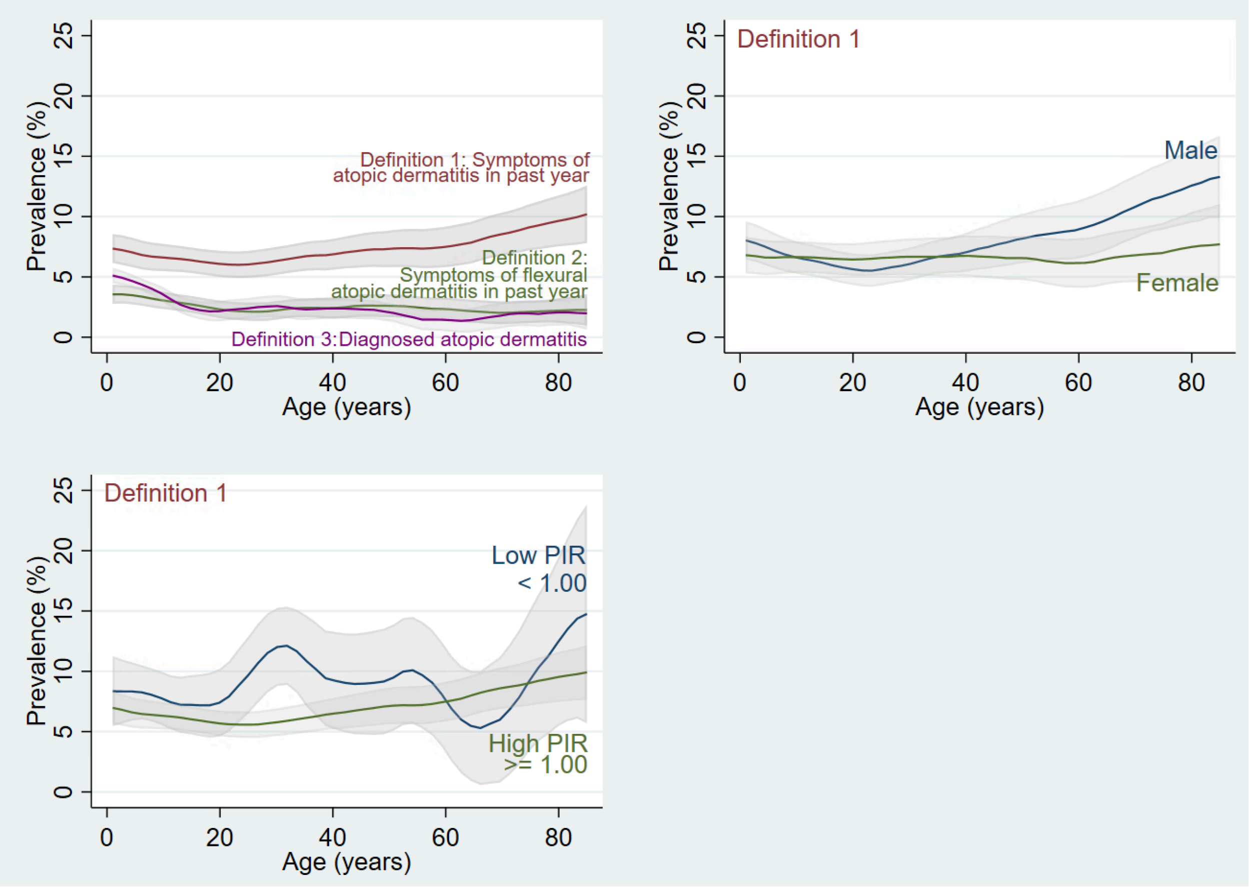

Supplement: S2 Fig — Local polynomial smoothed plots with shading indicating the 95% CIs generated from yearly cross-sectional calculations of the percent with prevalent atopic dermatitis during the past year from ages 0 to 85+. A) Three definitions representing prevalence of atopic dermatitis were formed by separate combinations of survey questions relevant to atopic dermatitis B) Prevalence of atopic dermatitis by sex using Definition 1 and C) Prevalence of atopic dermatitis by income using Poverty Income Ratio (PIR) using Definition 1. (PDF) [file pone.0258219.s002.pdf]
