# Supplementary material for: The epidemiology of atopic dermatitis in older adults: A population-based study in the United Kingdom
Source: PLoS One. 2021 Oct 6;16(10):e0258219. doi: 10.1371/journal.pone.0258219 (PMC8494374; doi:10.1371/journal.pone.0258219)

S3 Fig. Atopic dermatitis activity, by age group. Mean proportion of years with prevalent atopic dermatitis as a percent of total years of follow-up 95% confidence interval by age group. Primary analysis includes atopic dermatitis activity regardless of the number of years of follow-up. Sensitivity analysis excludes those with less than 2 years of follow-up time (total of 2.3% of individuals across all age groups).

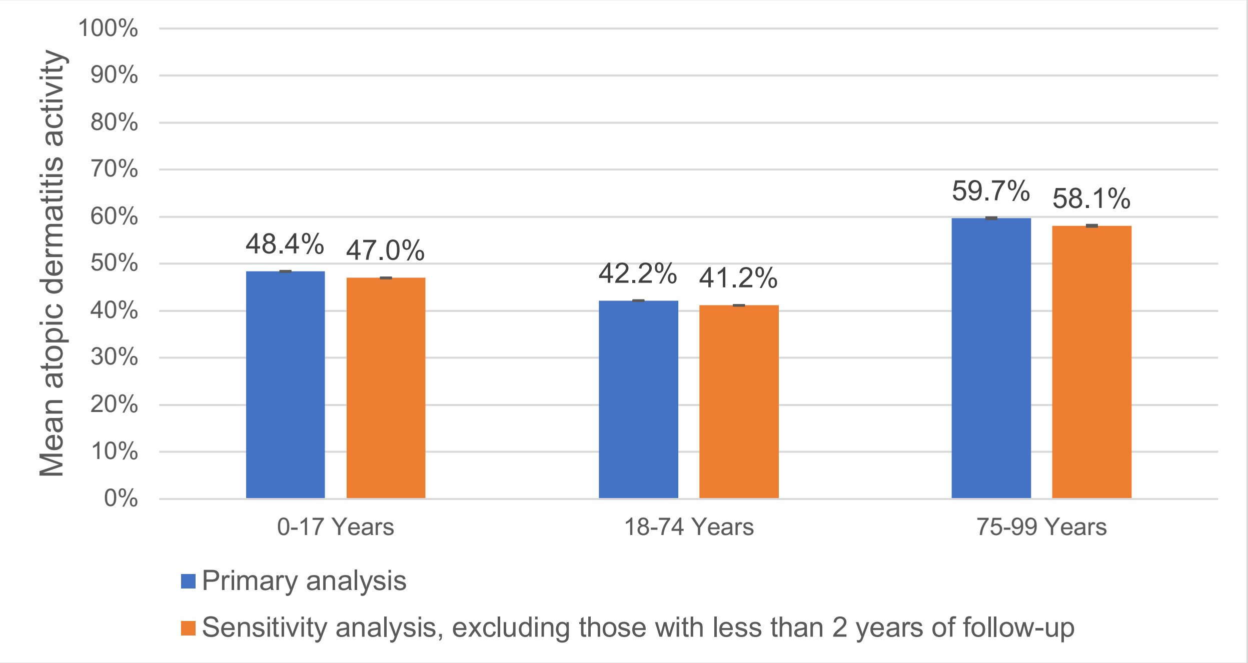

Supplement: S3 Fig — Mean proportion of years with prevalent atopic dermatitis as a percent of total years of follow-up 95% confidence interval by age group. Primary analysis includes atopic dermatitis activity regardless of the number of years of follow-up. Sensitivity analysis excludes those with less than 2 years of follow-up time (total of 2.3% of individuals across all age groups). (PDF) [file pone.0258219.s003.pdf]
