# Supplementary material for: The epidemiology of atopic dermatitis in older adults: A population-based study in the United Kingdom
Source: PLoS One. 2021 Oct 6;16(10):e0258219. doi: 10.1371/journal.pone.0258219 (PMC8494374; doi:10.1371/journal.pone.0258219)
Supplement: S1 Table — (PDF) [file pone.0258219.s004.pdf]

S1 Table. Atopic dermatitis diagnosis and therapy codes.

**READD Codes Defining Atopic Dermatitis**	
**Code**	**Description**
m111.00	Atopic dermatitis/eczema
m1120.0	Infantile eczema
m113.00	Flexural eczema
m11400	Allergic/intrinsic eczema
m12z100	Eczema NOS
**British National Formulary Codes**	
**Code**	**Description**
08.01.03.00	Antimetabolites and immune suppressants (methotrexate)
08.02.01.00	Antiproliferative immune suppressants (azathioprine, mycophenolate)
08.02.02.00	Other immune suppressants (cyclosporin, tacrolimus, corticosteroids)
13.00.00.00	Skin
13.01.00.00	Management of skin conditions
13.01.01.00	Vehicles
13.02.00.00	Emollient and barrier preparations
13.02.01.00	Emollient skin preparations
13.02.01.01	Emollient bath additives and shower preparations
13.02.01.02	Emollient skin preparations
13.02.02.00	Barrier preparations
13.03.00.00	Topical local anesthetic and antipruritics
13.04.00.00	Topical corticosteroids
13.05.00.00	Preparations for eczema and psoriasis
13.05.01.00	Preparations for eczema
13.05.03.00	Drugs affecting the immune response
13.05.03.00	Drugs affecting the immune response
13.10.01.01	Antibacterial preparations for skin
13.10.01.02	Antibacterial preparations for skin
13.10.03.00	Antiviral preparations for skin
14.05.00.00	Interferon gamma
**READ Phototherapy Codes**	
**Code**	**Description**
863..11	Phototherapy
8631	Phototherapy
8632.11	PUVA/phototherapy
8632.14	UVB phototherapy
8632.15	Ultraviolet light phototherapy
7G0E.00	Phototherapy to skin
7G0E200	Combined photochemotherapy and UVA light therapy to skin
7G0E300	Combined photochemotherapy and UVB light therapy to skin
7G0Ey00	Other specified phototherapy to skin
7G0Ez00	Phototherapy to skin NOS
863..00	Phototherapy/radiation therapy
863Z.00	Radiation/phototherapy NOS
Z6E1.12	Ultraviolet light phototherapy
Z6E1111	UVA phototherapy
Z6E1211	UVB phototherapy
Z6E1411	PUVA phototherapy
Z6E2.00	Photochemotherapy
Z6E5.00	Intermittent phototherapy
