# Supplementary material for: The epidemiology of atopic dermatitis in older adults: A population-based study in the United Kingdom
Source: PLoS One. 2021 Oct 6;16(10):e0258219. doi: 10.1371/journal.pone.0258219 (PMC8494374; doi:10.1371/journal.pone.0258219)
Supplement: S2 Table — (PDF) [file pone.0258219.s005.pdf]

S2 Table. Proportion of missing data for setting by age group.

Setting	Atopic dermatitis		Non-atopic dermatitis		Total
	Missing, frequency (row %)	Not missing, frequency	Missing, frequency (row %)	Not missing, frequency	
Children (0-17 years)	46,705 (18.27)	208,917	255,679 (22.36)	889,053	1,400,354
Adults (18-74 years)	90,608 (17.39)	429,794	1,326,740 (21.36)	4,884,536	6,731,678
Older adults (75-99 years)	16,811 (14.04)	101,619	178,476 (19.73)	725,998	1,022,904
Total	154,124 (17.20)	740,330	1,760,895 (21.32)	6,499,587	9,154,936
