# Supplementary material for: The epidemiology of atopic dermatitis in older adults: A population-based study in the United Kingdom
Source: PLoS One. 2021 Oct 6;16(10):e0258219. doi: 10.1371/journal.pone.0258219 (PMC8494374; doi:10.1371/journal.pone.0258219)
Supplement: S3 Table — (PDF) [file pone.0258219.s006.pdf]

S3 Table. Proportion of missing data for Townsend Score by age group.

Townsend Score	Atopic dermatitis		Non-atopic dermatitis		Total
	Missing, frequency (row %)	Not missing, frequency	Missing, frequency (row %)	Not missing, frequency	
Children (0-17 years)	17,902 (7.00)	237,720	99,619 (8.70)	1,045,113	1,400,354
Adults (18-74 years)	30,618 (5.88)	489,784	532,242 (8.57)	5,679,034	6,731,678
Older adults (75-99 years)	6,122 (5.17)	112,308	69,049 (7.63)	835,425	1,022,904
Total	54,642 (6.11)	839,812	700,910 (8.49)	7,559,572	9,154,936
