# Supplementary material for: The epidemiology of atopic dermatitis in older adults: A population-based study in the United Kingdom
Source: PLoS One. 2021 Oct 6;16(10):e0258219. doi: 10.1371/journal.pone.0258219 (PMC8494374; doi:10.1371/journal.pone.0258219)
Supplement: S4 Table — (PDF) [file pone.0258219.s007.pdf]

S4 Table. Mixed effects logistic regression results for complete case analysis and multiple imputation in random 1% sample of population

Variable	Age Group					
	**0-17 Years (N=161,208)**		**18-74 Years (N=570,479)**		**75-99 Years (N=51,039)**	
	AOR (95% CI) from regression model					
	**Complete Case**	**Multiple Imputation**	**Complete Case**	**Multiple Imputation**	**Complete Case**	**Multiple Imputation**
Age[a]	0.85 (0.85, 0.86)	0.86 (0.86, 0.87)	1.03 (1.03, 1.03)	1.03 (1.03, 1.03)	1.06 (1.04, 1.08)	1.06 (1.04, 1.08)
Calendar year at end of follow-up[a]	1.12 (1.10, 1.15)	1.12 (1.10, 1.14)	1.07 (1.05, 1.08)	1.07 (1.06, 1.09)	1.08 (1.04, 1.13)	1.09 (1.04, 1.14)
Sex						
Male	Reference					
Female	1.10 (0.94, 1.28)	1.06 (0.93, 1.22)	2.10 (1.84, 2.40)	2.06 (1.83, 2.33)	0.77 (0.54, 1.11)	0.78 (0.56, 1.09)
Setting						
Rural	Reference					
Urban	1.33 (1.09, 1.65)	1.26 (1.02, 1.57)	1.01 (0.85, 1.20)	1.02 (0.86, 1.21)	0.94 (0.61, 1.45)	0.94 (0.61, 1.46)
Townsend score						
Townsend 1 (least deprived)	Reference					
Townsend 2	0.95 (0.75, 1.19)	0.88 (0.71, 1.10)	0.84 (0.70, 1.02)	0.82 (0.68, 0.98)	0.76 (0.45, 1.27)	0.67 (0.41, 1.10)
Townsend 3	1.04 (0.83, 1.30)	1.00 (0.80, 1.25)	0.89 (0.73, 1.08)	0.89 (0.74, 1.07)	0.64 (0.37, 1.09)	0.68 (0.41, 1.12)
Townsend 4	1.01 (0.80, 1.27)	0.98 (0.79, 1.21)	0.96 (0.78, 1.17)	0.95 (0.78, 1.14)	1.13 (0.67, 1.90)	1.04 (0.63, 1.72)
Townsend 5 (most deprived)	0.99 (0.77, 1.28)	0.90 (0.71, 1.14)	0.94 (0.74, 1.18)	0.90 (0.73, 1.11)	1.05 (0.56, 1.97)	1.06 (0.68, 1.64)

Note. Each column represents a separate multivariable mixed effects logistic regression model.

AOR = adjusted odds ratio.

[a] Modeled as a continuous variable, value represents adjusted odds of atopic dermatitis for each additional year of age or each additional calendar year, respectively.
