# Supplementary material for: The epidemiology of atopic dermatitis in older adults: A population-based study in the United Kingdom
Source: PLoS One. 2021 Oct 6;16(10):e0258219. doi: 10.1371/journal.pone.0258219 (PMC8494374; doi:10.1371/journal.pone.0258219)
Supplement: S5 Table — (PDF) [file pone.0258219.s008.pdf]

S5 Table. Mean percent of atopic dermatitis across age groups by 5-year time spans from 1994-2013.

Mean % with disease	Age Group			
Calendar Year	0-17 Years	18-74 Years	75-99 Years	Total
1994-1997	6.6%	3.1%	5.6%	3.9%
1998-2002	7.6%	3.5%	6.8%	4.6%
2003-2007	9.0%	4.0%	8.5%	5.3%
2008-2013	8.9%	4.0%	8.9%	5.4%
Beta and p-value from linear test for trend[1]	ß=0.008, p=0.066	ß =0.003, p=0.052	ß =0.012, p=0.021	ß =0.005, p=0.044

Notes. [1]To estimate whether there was a linear trend by calendar year, we ran linear regression models (y=b (Calendar Year) + constant). ß represents the increase in the proportion of individuals with atopic dermatitis for each subsequent 5-year span.
