# Supplementary material for: The epidemiology of atopic dermatitis in older adults: A population-based study in the United Kingdom
Source: PLoS One. 2021 Oct 6;16(10):e0258219. doi: 10.1371/journal.pone.0258219 (PMC8494374; doi:10.1371/journal.pone.0258219)
Supplement: S1 Methods — (PDF) [file pone.0258219.s009.pdf]

S1 Supplemental Methods

Socioeconomic Status in the NHANES Validation Analysis

Poverty income ratio (PIR) was used in this validation analysis as a measure of socioeconomic status to parallel Townsend score in THIN. PIR is the ratio of a family's self-reported income to their appropriate poverty threshold based on the number and age of family members, as determined by the US Census Bureau. Individuals with a PIR value less than 1.00 are below the official poverty threshold and considered to be in poverty, while those with a PIR value greater than or equal to 1.00 are not considered to be in poverty.[1]

References

1. How the Census Bureau Measures Poverty [Internet]. [cited 2020 Aug 27]. Available from: https://www.census.gov/topics/income-poverty/poverty/guidance/poverty-measures.html
